# Errors in Results, Table 2, and Supplement 2

**DOI:** 10.1001/jamanetworkopen.2026.37618

**Published:** 2026-09-10

In the original article titled, “Octenidine or Sterile Water Cleansing and Late-Onset Sepsis in Neonates in the NICU: A Randomized Clinical Trial,”^1^ published July 15, 2026, there were errors in relative risk and 95% CI data. In the Results section, the 95% CI for the absolute risk difference for late-onset sepsis between the octenidine group and sterile water groups should have been “(95% CI, −4.1 to 7.6 percentage points)” rather than “(95% CI, 4.0 to 8.0 percentage points).” In the Results section and Table 2, for the intention-to-treat analysis the risk ratio for all-cause mortality in the octenidine group vs the sterile water group should have been 2.38 (95% CI, 0.85-6.67) rather than 1.00 (95% CI, 1.00-1.10); in Table 2 for the same comparison, the per-protocol analysis risk ratio should have been 2.40 (95% CI, 0.90-6.50) rather than 1.00 (95% CI, 1.00-1.10). Additionally, eFigure 4 in Supplement 2 has been updated. This article has been corrected.^1^
